# Mobile Apps for Oral Health Promotion: Content Review and Heuristic Usability Analysis

**DOI:** 10.2196/11432

**Published:** 2018-09-04

**Authors:** Brooks Tiffany, Paula Blasi, Sheryl L Catz, Jennifer B McClure

**Affiliations:** ^1^ Kaiser Permanente Washington Health Research Institute Seattle, WA United States; ^2^ Betty Irene Moore School of Nursing University of California, Davis Sacramento, CA United States

**Keywords:** oral health, oral hygiene, dental care, mobile health, eHealth, review

## Abstract

**Background:**

There has been an increase in consumer-facing mobile health (mHealth) apps in recent years. Prior reviews have characterized the availability, usability, or quality of popular mHealth apps targeting a range of health behaviors, but none has examined apps that promote better oral health care. Oral disease affects billions of people worldwide and mobile phone use is on the rise, so the market for well-designed and effective oral health apps is substantial.

**Objective:**

We examined the content and usability of popular oral health promotion apps to better understand the current state of these self-help interventions and inform the need and opportunity for future app development.

**Methods:**

Between February and March 2018, we identified oral health-focused apps that were designed for Android or iOS, available in English, and targeted adult consumers (as opposed to children or dental health professionals). The sample was limited to the most popular and highly rated apps on each platform. For each app reviewed, we assessed its basic descriptive characteristics (eg, platform, cost), evidence of a theoretical basis or empirical validation, key program functionality, and the extent to which the app addressed diet and tobacco and alcohol use as risk factors for oral disease. We characterized the framing (ie, gain vs loss) of all persuasive messaging and conducted a heuristic analysis to assess each app’s usability as a persuasive health technology.

**Results:**

Thirty-three apps were eligible for review based on the selection criteria. Two-thirds (22/33, 67%) were geared toward the general public as opposed to dental clinic patients, insurance plan members, or owners of specific electric toothbrushes. Most (31/33, 94%) were free to download, and a majority (19/33, 58%) were sponsored by software developers as opposed to oral health experts. None offered any theoretical basis for the content or had been empirically validated. Common program features included tools for tracking or reminding one to brush their teeth and assistance scheduling dental appointments. Nineteen apps (58%) included educational or persuasive content intended to influence oral health behavior. Only 32% (6/19) of these included a larger proportion of gain-framed than loss-framed messaging. Most of the apps did not mention diet, alcohol or tobacco—important risk factors for oral disease. Overall, the apps performed poorly on standard usability heuristics recommended for persuasive health technologies.

**Conclusions:**

The quality of the reviewed apps was generally poor. Important opportunities exist to develop oral health promotion apps that have theoretically grounded content, are empirically validated, and adhere to good design principles for persuasive health technologies.

## Introduction

Untreated oral conditions, including dental caries, severe periodontitis, and edentulism, affect about 3.5 billion people worldwide [1]. Oral health is integral to overall health [2]; oral disease contributes to unnecessary pain and suffering and is the fourth most costly disease to treat in most industrialized countries [3]. Routine oral hygiene, including daily brushing and flossing, is important for preventing oral disease and maintaining good oral health [4,5]. Routine dental visits are also important for maintaining good oral health because dentists can check for early signs of oral disease, provide teeth cleaning, and offer counseling about oral health behaviors [6]. Thus, promoting better oral health care is an important public health goal.

We believe mobile phones offer a promising strategy for reaching the public to deliver low-cost oral health promotion apps. According to a 2018 survey by the Pew Research Center, 77% of US residents now own a mobile phone [7]. Across 40 countries, a global median of 43% of residents owned a mobile phone in 2015, and mobile phone ownership is growing rapidly in countries with emerging and developing economies [8], making this technology a viable platform for direct-to-consumer public health interventions.

Along with the growth in mobile phone usage, there has been rapid growth in consumer-facing health promotion apps [9]. About 29% of those who have downloaded an app to a mobile phone or tablet report downloading a health-related app [10], and in 2017, there were 325,000 health-related apps available for download [11]. Mobile phone apps have been used to promote a variety of healthy behaviors including tobacco cessation [12], diabetes self-management [13], diet and nutrition [14], and physical activity [14].

Prior reviews have examined the efficacy of mobile health (mHealth) apps for behaviors such as weight loss and physical activity [15], self-management of chronic conditions [16], and identifying skin cancers [17]. Other reviews have sought to characterize the availability, usability, or quality of popular mHealth apps for common health issues such as supporting care during pregnancy [18], promoting physical activity [19] and encouraging smoking cessation [12]. But very little is known about the state of publicly available mHealth apps for adult oral health promotion. In a review of the literature, we only identified 1 cross-sectional survey of people who had used an app (Brush DJ) to improve their oral health self-care [20] and no prior reviews of the overall content, usability, or quality of existing oral health apps for adults. However, research has shown that text messaging interventions are associated with improvements in tooth brushing frequency [21], reducing plaque [22], and changing one’s oral hygiene index and gingival index [23], so it is plausible that well-designed oral health apps could be effective at improving knowledge, self-care behaviors, or facilitating receipt of needed professional dental care among adults.

To address the gap in the literature and inform future oral health intervention development, we conducted a systematic review of the most popular oral health apps available for Android and iOS phones. To do this, we sought to review the content and assess the usability of each app. Our content review included an examination of the basic features and functionality, inclusion of key oral health discussion topics, analysis of message framing, and assessment of whether the developers cited a theoretical or empirical basis for the content. The findings from this comprehensive review inform the overall quality of popular oral health promotion apps and speak to both the need and opportunity for additional mHealth intervention development.

## Methods

### Sample

We identified oral health-focused apps in the App Store and Google Play between February and March 2018. Apps were identified using the following search phrases: *oral health*, *dental health*, *teeth health*, *tooth health*, *mouth health*, *dental care*, *teeth care*, *and oral care*. The initial search revealed 1975 Android and 1005 iOS apps. To narrow the field, we excluded apps that were not in English and those designed specifically for dental health professionals, dental students, patients of specific dental practices, or children. We then restricted the sample to the most popular available for each platform based on the assumption that these were likely the highest quality and most frequently used apps. As such, we believe these represent a reasonable sample for evaluating the content and quality of current adult-focused oral health apps. A similar strategy has been used by others to limit the number of apps reviewed when it was not feasible to review all available apps [24]. We did not eliminate any apps that met our inclusion criteria based on their perceived quality.

To limit the search to those most popular, we followed the example of Abroms et al [24] and selected Android apps that had been installed at least 1000 times (n=24). Of these, 12 were only available for Android and 10 were also available for iOS. Similar download information was not available from the App Store, but according to Apple Inc, presentation order is correlated with user ratings, reviews, and user downloads [25], even though ratings are not always published, so we selected the first 12 iOS apps (excluding duplicates also found for Android), allowing a balance between Android-only and iOS-only apps. However, 1 iOS app was not compatible with iOS 11, so we were unable to include it in our final review. Thus, the final sample included 33 apps (12 for Android, 11 for iOS, and 10 for both Android and iOS). Those available on both platforms were reviewed through Android since the Google Play store provided more complete information about each app than was available at the App Store. Several were available to the public but included content that was restricted to consumers who had purchased electric toothbrushes sold by the app sponsor. For these, we only reviewed the free-to-access content. All apps were reviewed on tablet devices.

### Content Review: Key Features, Functionality, Content Basis, and Messaging

For each app we documented the developer/sponsor, platform, cost, number of installations, and user ratings. We summarized the basic functionality and noted whether the app or its descriptive summary at the app store indicated that it had been empirically validated. We also conducted a literature review to identify empirical evaluations that might be included in the list. Next, we documented whether each app or its descriptive summary included any reference to a theoretical or empirical basis for the educational or persuasive content.

In addition, we characterized the framing of all persuasive health messages used. According to prospect theory, potential losses are more motivating than potential gains when risky actions are being considered, but gains are more motivating than losses for low-risk behaviors [26]. This theory is commonly applied when writing persuasive health messages. In fact, prior research has shown that gain-framed messages are particularly effective for promoting oral hygiene behaviors [27-31]. Gain-framing highlights the benefits of action, such as healthier teeth and gums or fresh breath. In contrast, loss-framed messages highlight the risks of inaction, such as increased cavities or oral disease. Thus, we were interested in understanding the extent to which this strategy was being used in each app. To assess framing, we captured screenshots of all content and then systematically reviewed and coded all educational or persuasive written messages. Messages were deemed gain-framed if they emphasized the benefits of positive oral health behaviors like brushing and flossing. Messages were deemed loss-framed if they emphasized the risks of not engaging in positive health behaviors. Messages that were neither gain- nor loss-framed were coded as neutral.

Because diet and tobacco and alcohol use are important risk factors for oral disease [32], we noted whether each topic was discussed (yes/no) and characterized the level of discussion as either brief (ie, up to a few sentences), partial (ie, a subsection dedicated to this topic, such as a short paragraph), or full (ie, 1 or more full sections dedicated to the topic). We then summarized the key talking points of this discussion to assess the quality and relevance of the content. In particular, we were interested in whether each discussion identified diet or alcohol or tobacco use as a risk factor for oral disease.

All content was reviewed and characterized using the methods outlined above by the first author (BT). A second reviewer (JM) reviewed the summarized findings and validated the first author’s characterization of key content elements including message framing, level of discussion focused on key topics such as tobacco and alcohol, and whether the content linked diet, alcohol, or tobacco to oral disease risk.

### Heuristic Analysis

Each of the apps reviewed is an example of a persuasive health technology [33], intended to change users’ attitudes and/or behavior. The effectiveness of a persuasive technology is not limited to the quality of its written content; it is also affected by the app’s user interface and functionality. If the app is difficult to operate, consumers will stop using it, and as a result the design will undermine the app’s intended effects. Thus, it is essential that persuasive technologies comply with basic best practice design principles.

To further assess the quality of the selected apps, we reviewed and rated each based on 10 heuristics recommended for improving the usability of persuasive health technologies [34]. Kientz et al [34] argue that these heuristics are the most appropriate for technologies designed to persuade the end user to be healthier, and her team has found that they identify severe usability problems in persuasive technologies more frequently than the general heuristics proposed by Nielsen [35]. The heuristics recommended by Kientz et al [34] are as follows:

- Appropriate functionality: technology should meet usability, mobility, visibility, and durability needs according to the settings in which it might be used. The technology should function effectively in the user’s environment by being easy to use and integrate into one’s daily life and routine.
- Not irritating or embarrassing: technology should not irritate or embarrass the user, even after using the product repeatedly and regularly over a long period of time. This relates to aspects such as the presence of the product itself in the user’s environment; the degree to which the technology intrudes upon the user’s daily life; the timing, type, accuracy, and amount of feedback given; and the capability for customized settings and privacy controls.
- Protects user’s privacy: system allows users to keep personal information private. Users can control what, when, to whom, and how much information is made public. Any public information is kept abstract.
- Use of positive motivation strategies: technology recognizes when target behaviors have been performed or goals have been met and uses positive reinforcement strategies to promote continued progress. App avoids use of punishment for failure to perform target behaviors or meet goals.
- Usable and aesthetically appealing design: visual design of the technology is attractive and appealing and adheres to basic usability standards. Design captures and sustains the user’s interest, enhances user engagement with the technology, and adds to the credibility and usability of the product.
- Accuracy of user information: technology should not inaccurately record or misrepresent the user’s behavior (for instance, due to limitations in automatic sensing capabilities or the inability to use the device in certain environments). If necessary—to obtain an accurate, comprehensive account of behavior—the technology should allow users to edit data records and/or manually input additional data that the device is incapable of detecting automatically.
- Appropriate time and place: information, feedback, and assistance are provided at an opportune time and place (ie, when and where it is needed, at the most appropriate time, and in the most effective manner).
- Visibility of user’s status: technology should always keep the user informed about progress toward goals through appropriate feedback within a reasonable time frame. Feedback is accurate and easily understood (eg, through use of abstract displays, summary data).
- Customizability: users should be able to customize aspects of the technology, for example, creating personalized goals and customizing product settings (public/private data, interface, etc). However, customizability should not interfere with persuasive aspects.
- Educate users: users should understand why their actions promote positive behaviors and how their goals are being met. This includes which specific behaviors lead to the accomplishment of a larger goal. The technology should engage users in an active process whereby they learn information and gain skills relevant to their goals, particularly skills that would enable them to continue to progress toward goals even in the absence of the technology.

**Table 1:** Best and worst case examples of usability by heuristic domain.

Heuristic	Best case	Worst case
Appropriate functionality	Has a comprehensive set of persuasive health features such as a toothbrush timer, goal tracking, and reminders that run smoothly Is intuitive and easy to use Free of errors and crashes	Primary function is a toothbrush timer, which doesn’t work Lacks basic features such as reminders, sound controls, etc. Has frequent errors and crashes repeatedly
Not irritating or embarrassing	Free of ads and other unnecessary interruptions Functions smoothly, navigation is intuitive, and screens and features load quickly Gives user the ability to customize settings and controls such as sound, timer length, and privacy	Frequently interrupts user with full-screen ads, flashing banners, and solicitations Slow loading times; disorganized, inconsistent navigational controls No user guides, assistance, or help of any kind provided
Protects user’s privacy	Log-in/password protected Informs/reassures user about privacy protections and rights Allows user to control their data permissions/usage	No log-in/password option No mention of privacy policy or what happens to data Shares data publicly or with third parties such as advertisers
Use of positive motivation strategies	Primarily uses gain-framed messaging and positive imagery throughout Positively reinforces achievement of target behavior: “Great job! Keep it up!” Rewards user with badges, points, or other incentives when achieving target behaviors/goals	No positive motivation strategies are used at all; says nothing when you complete a tooth brushing session Primarily uses loss-framed messaging and negative imagery (eg, cartoon of person suffering from bad breath) Warns potential mates will run from you if you have bad breath
Usable and aesthetically appealing design	Interface is well designed and professional looking High-quality, attractive images and graphics are used Navigational elements and controls are intuitive and easy to use, keep the user well oriented	Unappealing color choices, such as a puke-green background Poor image quality; pixelated, unattractive imagery and graphics; reuses the same image for different sections Disorganized, disorienting, and inconsistent navigation controls
Accuracy of user information	Option to track multiple aspects of user information and behavior Tracking features are accurate, editable, and function appropriately Allows user to manually input, edit, or delete their information	Has no ability to track or store user information/behavior Tracking features do not function properly or are highly inaccurate Does not provide a way to input, edit, or delete existing user information
Appropriate time and place	Information is coherent and well organized for consumption Provides user with mini-tutorials when accessing new features Simply shaking the device allows the user to easily report an issue	Textual information is plagued by spelling, grammatical, or syntax errors; difficult to consume No guides, assistance, or help of any kind is provided for the user App randomly asks user to solve math problems
Visibility of user’s status	Provides summary data of user’s stats on home screen Provides more in-depth explanation of user progression data with historical graphs/charts Shows progression bars as user works toward goals	No user data is tracked, so there is no feedback on status or progress towards goals Does not let the user know which content they’ve reviewed; doesn’t provide option to do so Toothbrush timer is tiny; difficult to see progress
Customizability	Provides ability to customize colors, sounds, and notifications Provides ability to choose from among several oral hygiene goals Provides ability to create multiple user profiles so a family can share the device, for instance	No ability to customize colors, sounds, or notifications No ability to set personalized goals or tailor oral hygiene strategies, such as timer length No ability to customize privacy settings or control user data
Educates users	Provides educational material on the benefits of oral hygiene practices Provides videos that explain how to properly brush, floss, and perform other types of oral care Positively reinforces target behaviors by reminding user of end-goal benefits	No educational, instructional, or persuasive content whatsoever No clear goals for the user to achieve Does not attempt to engage the user in any process or explain why user should perform certain types of behaviors

For each of the heuristics above, we applied a standard severity scoring system recommended by Nielsen [36] and adopted by Kientz [34]; however, we modified our application of the scoring system for the purposes of this paper. Rather than scoring each identified usability problem, we assigned a single severity score for each heuristic domain for each app and then calculated an overall severity score for each app. This methodology allowed us to better compare the usability of the selected apps to one another and comment on the overall user-centeredness and quality of the apps. The scoring criteria applied were as follows: 0=not a usability issue, 1=cosmetic problem only, 2=minor usability problem, 3=major usability issue, and 4=usability catastrophe. We took the perspective that an ideal persuasive technology should adhere to the objectives of all 10 heuristics. If a domain was not addressed at all, it was coded as a usability catastrophe. Due to resource limitations, a single reviewer (BT) coded each app, but the application of the scoring system was discussed and agreed upon by all coauthors and individual items were randomly selected and reviewed by the team to ensure a consistent application of criteria.

Table 1 includes specific examples of how the scoring criteria were applied, illustrating the type of characteristics observed in the reviewed apps that would receive a low severity score (best case) and a high severity score (worst case).

## Results

### Basic Features and Functionality

A total of 33 apps met the selection criteria and were reviewed (Table 2). Most were sponsored by a software developer (19/33, 58%) or dental care provider (6/33, 17%) and were targeted to the general public (22/33, 67%). However, 12% (4/33) were offered by companies as an adjunct for their electric toothbrushes. All but 2 apps (31/33, 94%) were free to download.

Common design features included the ability to provide feedback on the app (12/33, 36%), customize aspects of appearance or sounds (12/33, 36%), set up a log-in account (9/33, 27%), access customer support (9/33, 27%), customize one’s oral health goals (8/33, 24%), and share progress with others (5/33, 15%). Four apps (12%) specifically promoted oral hygiene products for purchase; 6 included gamification features such as awarding badges for behavioral milestones and accomplishments.

Key functions are presented in Table 3. Most (20/33, 61%) included a timer for brushing teeth, and for 45% (15/33) of the apps reviewed, this was the primary function of the app. Other common functions included tips for better managing oral hygiene, reminders and alerts for improving oral hygiene (eg, notification to floss before bed or replace an old toothbrush), and general oral health-related educational content. Five apps (15%) allowed people to communicate with a dental health professional to either ask questions, request a video-based oral health assessment, or inquire about dental insurance. Five (15%) were designed to help users look for a dental care provider.

### Theoretical or Empirical Basis

None of the apps cited a theoretical foundation for design or content. Only 1 mentioned any evaluation of the app’s impact on changing users’ knowledge, attitudes, or behavior. The cited study, however, was limited to a cross-sectional survey of users’ perceptions and did not assess the actual effectiveness of the intervention in a randomized or longitudinal study [20]. None of the apps appeared to have been empirically validated.

### Key Messaging: Content and Framing

More than half (19/33, 57%) of the apps included written content intended to influence oral health behavior as opposed to simply providing instructional directions for how to use the features or functionality (eg, toothbrush timer). The majority of these apps (15/19, 79%) included a mix of gain- and loss-framed messages (Table 4). A third (6/19, 32%) included a larger proportion of gain-framed messages compared to loss-framed messages, and 1 app included only neutral messaging with neither a gain nor loss frame.

Examples of gain-framed messages included:

To keep your teeth and gums healthy you should clean between teeth daily with floss or an interdental cleaner.

Brush your way to a fresh smile.

Examples of loss-framed messages included:

In general, the higher the frequency and quantity of sugary foods and drinks you intake per day, the more at risk you are of developing tooth decay.

If you don’t floss and brush your teeth regularly, any food trapped between your teeth will be broken down by the bacteria and may be responsible for bad breath.

Twelve apps (36%) included some discussion of diet, but the discussion was either brief (5/12, 42%) or partial (5/12, 42%). Only 2 provided more comprehensive dietary information. Most of the apps that mentioned diet (7/12, 58%) recommended limiting or avoiding sugary foods that cause tooth decay or bad breath, and 33% (4/12) mentioned that acidic food or drinks can damage teeth. Several apps (3/12, 25%) endorsed eating a diet rich in fruits and vegetables, but 1 suggested eating fruits and vegetables could stain one’s teeth.

Eleven apps (33%) briefly or partially discussed tobacco use. None included a full discussion of the effects of tobacco on oral health outcomes. Five of the 11 apps that included some tobacco discussion (45%) advised people not to use or to quit tobacco, 36% (4/11) emphasized tobacco’s role in causing bad breath, 36% (4/11) emphasized that tobacco causes tooth stains, and 36% (4/11) linked tobacco use to oral disease. None referred people to stop-smoking treatment services.

Nine apps (27%) briefly addressed alcohol use. Seven of these (7/9, 78%) suggested drinking be limited or avoided to reduce the risk of unwanted oral health issues such as bruxism, bad breath, dry mouth, tooth decay, or stained teeth. Only 1 app noted that alcohol use is a risk factor for oral cancer. None referred people to treatment services to reduce their drinking.

**Table 2:** Reviewed apps.

Name	Platform	Sponsor	Cost	Number of installs^a^	Number of user ratings^a^	Average user rating^a^
24/7 Live Dentist Response	iOS	Digital health company	Free	Unknown	Unknown	Unknown
Bad Breath	Android	App developer	Free	1000	3	3.7
Bad Breath Guide	iOS	Dental provider	Free	Unknown	Unknown	Unknown
Brush DJ	Android & iOS	App developer	Free	100,000	1207	4.1
Brushing and Whitening Teeth	Android	App developer	Free	5000	106	4.4
Brush'n'Save	Android	Dental provider	Free	10,000	74	4
Brushy	Android	App developer	Free	10,000	115	3.3
Colgate Connect	iOS	Oral hygiene product company	Free	Unknown	81	4.3
DDS Anywhere	Android & iOS	App developer	Free	5000	23	4
Delta Dental	Android & iOS	Insurance provider	Free	100,000	793	3
Dentacare - Health Training	Android	App developer	Free	10,000	Unknown^b^	Unknown^b^
DentAdvisor: Oral Care Expert	iOS	App developer	$1.99	Unknown	Unknown	Unknown
Dental Care	Android	App developer	Free	1000	9	4.6
Dental Care—Target Smile	Android	App developer	Free	1000	40	4.9
Dental Desk	Android	Dental provider	Free	10,000	66	4.6
Do I Grind	Android & iOS	Digital health company	Free	1000	40	4.1
FoodForTeeth^c^	iOS	Dental provider	Free	Unknown	Unknown	Unknown
Healthy Teeth^d^	iOS	App developer	Free	Unknown	Unknown	Unknown
Kolibree	Android & iOS	Oral hygiene product company	Free	5000	136	3.3
Let’s Brush Free	iOS	App developer	Free	Unknown	Unknown	Unknown
Moment of Tooth	iOS	App developer	Free	Unknown	8	4.9
My Dental Care	Android & iOS	Dental provider	Free	500	14	5
MySmile	Android & iOS	App developer	Free	10,000	126	4.1
Oral-B App	Android & iOS	Oral hygiene product company	Free	1,000,000	12,489	3.3
Philips Sonicare	Android & iOS	Oral hygiene product company	Free	50,000	564	2.7
QuickBrush Toothbrush Timer	Android	App developer	Free	10,000	176	4.2
Smile—Dental Hygiene Analysis	iOS	Dental provider	Free	Unknown	14	3.6
Tooth Notes	iOS	App developer	$0.99	Unknown	Unknown	Unknown
Toothbrush Pacer	Android	App developer	Free	10,000	98	4
Toothbrush timer	Android	App developer	Free	100,000	1046	3.5
Toothbrush Timer	Android	App developer	Free	50,000	776	3.8
Toothy: Brush Floss Rinse!	iOS	App developer	Free	Unknown	401	4.6
United Concordia Dental Mobile	Android & iOS	Insurance provider	Free	50,000	232	3.3

^a^Details on the number of installations, number of user ratings, and average rating are only available in the App Store for some iOS-based apps. Data presented on apps available for both Android and iOS list data from Google Play only. User ratings scored on a 1 (worst) to 5 (best) scale. All data were current as of the time of this review.

^b^This app was released as a beta version, so rating information was not yet being tracked.

^c^Full app name: FoodForTeeth—Food Database and Diet Diary.

^d^Full app name: Healthy Teeth—Tooth Brushing reminder with timer.

**Table 3:** Summary of key app functions.

Key function	Total, n (%)
Tooth brushing timer	20 (61)
Tips for better oral hygiene	16 (48)
Oral health educational content	13 (39)
Oral hygiene alerts or reminders	13 (39)
Tracking of oral health behaviors (eg, brushing)	9 (27)
Tracking of dental appointments	7 (21)
Ability to communicate with a dental professional	5 (15)
Ability to search for dentists	5 (15)

**Table 4:** Gain and loss framing in apps with persuasive health messages. Table does not include apps that provided instructional content only (n=14).

App name	Total messages, N	Gain framed, n (%)	Loss framed, n (%)	Neutral framed, n (%)
MySmile	22	0 (0)	9 (41)	13 (59)
Do I Grind	21	3 (14)	11 (52)	7 (33)
Delta Dental	81	8 (10)	30 (37)	43 (53)
Bad Breath	159	31 (20)	46 (29)	82 (52)
United Concordia Dental Mobile	113	7 (6)	18 (16)	89 (79)
Bad Breath Guide	352	48 (14)	74 (21)	220 (63)
Brushing and Whitening Teeth	424	9 (2)	16 (4)	98 (23)
Dental Desk	172	6 (4)	16 (9)	154 (90)
FoodForTeeth^a^	499	50 (10)	73 (15)	377 (76)
My Dental Care	691	45 (7)	70 (10)	576 (83)
DentAdvisor: Oral Care Expert	909	42 (5)	77 (9)	777 (86)
QuickBrush Toothbrush Timer	13	1 (8)	1 (8)	11 (85)
Brush DJ	19	0 (0)	0 (0)	19 (100)
Dental Care	430	69 (16)	65 (15)	295 (69)
Dental Care—Target Smile	48	1 (2)	0 (0)	47 (98)
Moment of Tooth	21	8 (38)	5 (24)	8 (38)
Let’s Brush Free	21	5 (24)	0 (0)	16 (76)
Philips Sonicare	11	8 (73)	1 (9)	2 (18)
Oral-B App	23	18 (78)	2 (9)	3 (13)

^a^Full app name: FoodForTeeth—Food Database and Diet Diary.

### Heuristic Review

Although not all the reviewed apps included explicit health persuasion messages, all were intended to influence users’ oral health attitudes and behavior. Thus, we reviewed all 33 apps using the heuristics recommended for persuasive health technologies.

Table 5 summarizes the number of apps that received each severity score by heuristic. Most apps received a severity rating of 3 or 4 across each heuristic. In general, apps scored poorly on protecting users’ privacy but had fewer issues with usability and aesthetic appeal. We also calculated an overall severity score for each app by summing the scores across all domains. Across all apps, the average overall severity score was 30 out of 40 (range 16 to 39). This score reflects major, systemic usability problems.

**Table 5:** Number of apps receiving usability severity scores for each persuasive health technology heuristic.

Heuristic	No issues (score=0)	Cosmetic issues (score=1)	Minor issues (score=2)	Major issues (score=3)	Usability catastrophe (score=4)
Appropriate functionality	0	0	5	26	2
Not irritating or embarrassing	0	1	9	20	3
Protects user privacy	2	0	4	5	22
Use of positive motivation strategies	0	3	1	17	12
Usable and aesthetically appealing design	0	6	13	12	2
Accuracy of user information	0	0	3	14	16
Appropriate time and place	0	4	2	25	2
Visibility of user status	0	4	1	20	8
Customizability	0	0	5	10	18
Educates users	0	0	4	20	9

## Discussion

### Principal Findings

We selected 33 of the most popular and highly rated oral health apps available for Android and iOS and reviewed the design, content, and usability of each. As a group, apps were of poor quality. All were intended to influence adults’ oral health attitudes and behaviors, but none were empirically validated to demonstrate their effectiveness. None cited any theoretical basis for the content, which is not unusual for mHealth apps, but since the majority were created by developers who did not appear to be affiliated with oral health or behavioral science experts, it is likely that the content and design of most were not driven by sound behavioral theory. As a case in point, prospect theory suggests that gain-framed messages are more effective at promoting preventive health behaviors than loss-framed messages [26,37]. This theory has some empirical basis, as prior research has shown gain-framed messages to be effective at promoting oral hygiene behaviors [27-31]. As such, one might expect to see gain-framing (as opposed to loss-framing) as a central feature in apps designed to improve oral hygiene behavior. However, of the 19 apps that included any explicit persuasive messaging, only 6 included content balanced in favor of gain-framed messaging and most of these still included loss-framed messages. Only 2 apps included gain-framed messaging without loss-framed messaging, but both included only a few gain-framed messages—far less than might be considered ideal for a persuasive intervention.

The oral health educational content had other issues, as well. For example, diet, tobacco, and alcohol are significant risk factors for oral disease [32], yet one-third or less of the apps reviewed discussed each of these important topics. When included, the content was typically brief and sometimes included information that might be considered counter-productive to the goal of improving users’ health behavior. A prime example is the app that advised users that eating fruits and vegetables can stain one’s teeth. While there is some evidence that eating certain fruits, vegetables, dairy, or soy products may contribute to the development of dark spots on the teeth called “black stain,” which are microflora deposits, the prevention or management of this condition is more complex than avoiding fruits and vegetables [38,39] and, in fact, the American Dental Association recommends that eating fruits and vegetables can benefit oral health [40]. In other examples, the developers’ attempts to use humor seemed to undermine the intended message. For instance, 1 app advised users that whether or when they clean their tongue should depend on whether they will be meeting “the crush of their life” and what they have eaten recently. In contrast, according to the American Dental Association, there is no evidence that brushing or scraping one’s tongue prevents bad breath [41].

It is also notable that of the apps that included any discussion of tobacco and alcohol, very few linked use of these substances to oral cancer risk. Most highlighted the cosmetic (eg, stained teeth) or social implications (eg, bad breath) of their use instead. Given the independent and synergistic effects that these substances have on oral disease [32], future oral health apps should more clearly articulate this risk and consider providing treatment referral information for those interesting in quitting or cutting back.

Finally, the reviewed apps all had significant usability issues based on our heuristic review. Only 2 took measures to protect users’ privacy, and none received a perfect severity score (ie, 0) on the other 9 heuristics. Significant usability issues (severity score of 3 or 4) were noted for most apps across the heuristic domains. Three apps sponsored by companies promoting oral health products (Oral B, Philips Sonicare, and Colgate Connect) were generally well designed and received higher scores but were narrowly intended to promote use of their products, resulting in lower overall scores as persuasive health technologies.

### Strengths and Limitations

This study has a number of notable strengths, including its novelty. To our knowledge, this is the first review of oral health promotion apps. As such, this paper addresses an important gap in the literature. It also establishes the need and opportunity to create high-quality oral health promotion apps targeting adults. Oral disease affects billions of people worldwide [1] and mobile phone use is rapidly expanding around the world [8], so the impact of effective oral health interventions delivered via mobile phone could be substantial.

Another strength of this review is its comprehensive nature, in which both the quality of persuasive health content and user-centered design of the apps were evaluated. Our application of Kientz’s persuasive health technology heuristics is also novel. Typically, these heuristics are used to identify a range of user issues and each issue is scored based on its severity rather than assigning a score to each heuristic domain or an overall severity score to the entire app. However, our approach was reasonable for the purposes of this review since it allowed us to compare the relative quality of the user design across apps using a common evaluation and scoring scheme.

Study limitations should also be noted. First, it is possible that there are existing oral health apps which are of higher quality than that of the most popular apps we reviewed. Resource restraints prevented us from reviewing all of the available apps (nearly 3000 were identified based on our keywords), but we believe our focus on the most popular apps is reasonable because these are the apps which are most frequently being used by the public. Next, the downside of our comparative heuristic review is that it treated each heuristic as equally important, which may not be the case. For this reason, we presented both overall app severity scores and domain-specific scores so readers can get a better sense of where usability issues were observed. But we acknowledge that depending on the nature of the app, performance in some heuristic categories may be more important than others so in future reviews it might be more appropriate to differentially weight the domains. Another limitation is that our heuristic review was performed by a single coder trained in user-centered design using standardized scoring criteria. More typically, if the goal had been to delineate all of the observed issues, a group of coders might be used. This was prohibited due to limited project resources but is also unlikely to have changed our conclusion about the quality of the design of these apps as a group based on the pervasiveness and severity of issues observed. Finally, we note that our review was limited to content that was available through installation of each app. Additional content only available to certain audiences such as health plan members or owners of purchased electric toothbrushes and protected via account log-in could not be viewed.

### Conclusions

Many oral health apps are available to consumers, but based on this review of the most popular and highly rated ones, the quality of these apps is generally poor. Important opportunities exist to develop oral health promotion apps whose content is theoretically grounded and evidence-based and that adhere to good design principles for persuasive health technologies.
